# Vascular Endothelial Dysfunction in β-Thalassemia Occurs Despite Increased eNOS Expression and Preserved Vascular Smooth Muscle Cell Reactivity to NO

**DOI:** 10.1371/journal.pone.0038089

**Published:** 2012-06-19

**Authors:** Ekatherina Stoyanova, Marie Trudel, Hady Felfly, Wafaa Lemsaddek, Damien Garcia, Guy Cloutier

**Affiliations:** 1 Laboratory of Biorheology and Medical Ultrasonics, University of Montreal Hospital Research Center, Montreal, Quebec, Canada; 2 Molecular Genetics and Development, Clinical Research Institute of Montreal, Montreal, Quebec, Canada; 3 Department of Radiology, Radio-Oncology and Nuclear Medicine; and Institute of Biomedical Engineering, University of Montreal, Montreal, Quebec, Canada; Maastricht University, The Netherlands

## Abstract

**Aims:**

The hereditary β-thalassemia major condition requires regular lifelong blood transfusions. Transfusion-related iron overloading has been associated with the onset of cardiovascular complications, including cardiac dysfunction and vascular anomalies. By using an untransfused murine model of β-thalassemia major, we tested the hypothesis that vascular endothelial dysfunction, alterations of arterial structure and of its mechanical properties would occur despite the absence of treatments.

**Methods and Results:**

Vascular function and structure were evaluated *ex vivo.* Compared to the controls, endothelium-dependent vasodilation with acetylcholine was blunted in mesenteric resistance arteries of β-thalassemic mice while the endothelium-independent vasodilator (sodium nitroprusside) produced comparable vessel dilation, indicating endothelial cell impairment with preserved smooth muscle cell reactivity to nitric oxide (NO). While these findings suggest a decrease in NO bioavailability, Western blotting showed heightened expression of aortic endothelial NO synthase (eNOS) in β-thalassemia. Vascular remodeling of the common carotid arteries revealed increased medial elastin content. Under isobaric conditions, the carotid arteries of β-thalassemic mice exhibited decreased wall stress and softening due to structural changes of the vessel wall.

**Conclusions:**

A complex vasculopathy was identified in untransfused β-thalassemic mice characterized by altered carotid artery structure and endothelial dysfunction of resistance arterioles, likely attributable to reduced NO bioavailability despite enhanced vascular eNOS expression.

## Introduction

β-thalassemia is an inherited hemoglobin disorder resulting from impaired production of β-globin chains of the hemoglobin tetramer. The resultant phenotype is chronic hemolytic anemia of varying severity, depending on the level of β-globin chain deficiency and subsequent α-globin chain accumulation. β-thalassemia major is characterized by severe transfusion-dependent anemia, starting from the first year of life, whereas β-thalassemia intermedia corresponds to a milder, generally transfusion-independent form with later clinical onset [1]. Transfusion therapy in β-thalassemia major patients requires adequate iron chelation treatments to avoid its progressive accumulation in several organs, evoking subsequent tissue damage and, eventually, death.

Although lifelong blood transfusions combined with adequate chelation therapy have significantly improved the survival of β-thalassemia major patients, cardiac complications remain the main cause of mortality in both β-thalassemia major and intermedia [1]–[3]. In addition, arterial and venous thromboembolic events in β-thalassemia major patients have been reported [4]. Several pathogenic factors contribute to these complications, including a chronic hypercoagulable state [5], increased erythrocyte aggregation [6] and endothelial adhesion of thalassemic erythrocytes in microvessels [7]. Furthermore, *in vivo* evidence of endothelial cell activation [8], [9] and impaired flow-mediated dilation in the brachial arteries of β-thalassemic patients [10], [11] implicate endothelial dysfunction in the pathogenesis of the above-mentioned vascular complications. Studies have revealed flow-mediated endothelial dysfunction in conduit arteries of optimally-chelated, transfusion-dependent β-thalassemic patients [10]–[12]. However uncertainty remains regarding the integrity of resistance artery endothelial vasomotor function. In addition, while these investigations have suggested decreased nitric oxide (NO) bioavailability, the underlying mechanisms of endothelial dysfunction and the specific role of enzymatic NO synthase (NOS) expression have yet to be elucidated.

Endothelial dysfunction generally leads to vascular remodeling [13], namely, arterial structural alterations and, consequently, potential changes in mechanical properties. In β-thalassemic patients, ultrasonographic measurements have demonstrated impaired elastic properties of the aorta [14], [15] and carotid arteries [11], suggesting reorganization of the vascular wall involving smooth muscle cells, elastin and collagen. Such a hypothesis, however, remains to be verified with experimental evidence and without the potential confounding impact of iron accumulation in vascular tissue.

Several β-thalassemic mouse models replicating phenotypic aspects and hematological anomalies of β-thalassemia major have been produced to characterize the pathogenesis and development of potential therapeutic strategies. To date, however, no studies have investigated vascular function and structure in β-thalassemic mice. In addition, the vasculature in β-thalassemia has not yet been examined without the confounding effects of transfusions and subsequent transfusional iron overload. The objectives of this study were to gain mechanistic insight into resistance artery vascular function by characterizing the NO-dependent endothelial vasodilatory function of isolated mesenteric arteries, and by evaluating carotid artery mechanical properties and structure in a non-transfused mouse model of β-thalassemia major.

## Methods

### Experimental Animals

Experimental procedures, including *ex vivo* sample analyses, were conducted in accordance with guidelines of the Institutional Animal Care Committee of the University of Montreal Hospital Research Center. The investigation conformed with guidelines of the Canadian Council on Animal Care and the Guide for the Care and Use of Laboratory Animals published by the US National Institutes of Health (NIH Publication No. 85-23, revised 1996, Assurance Number A5377-01).

Male homozygous β-thalassemic (homo-βthal, Hbb^d3(th)/d3(th)^) and bone marrow-transplanted control mice (Hbb^+/+^) were generated, as described previously [16]. Briefly, control C57BL/6J mice from Jackson Laboratories (Bar Harbor, ME) and β-thalassemic mice homozygous for deletion of the murine β-major gene (Hbb^d3(th)/d3(th)^) [17] were bred onto the C57BL/6J background for >16 generations. Bone marrow cells harvested from either homozygous β-thalassemic (Hbb^d3(th)/d3(th)^) or wild type C57BL/6J-*Gpi1^a^* (Hbb^+/+^) donors were injected (1.8×10^6^ cells) into sub-lethally-irradiated (8.75 Gy, Mark I-68A-1 Research Irradiator, San Francisco, CA), 2-month-old C57BL/6J-*Gpi1^b^* recipients. Bone marrow engraftment was evaluated in both mouse groups 2–5 months after transplantation. Only recipients displaying complete hematopoietic engraftment were included in the study, i.e. recipients with the sole expression of either hemoglobin minor for homo-βthal mice or the specific glucose phosphate isomerase isotype marker *Gpi1^a^* for the controls.

Hematocrit levels were quantified in 14-month-old mice immediately before sacrifice. The animals were euthanized by CO_2_ inhalation, and the entire intestine as well as the left common carotid artery were quickly dissected and placed in ice-cold physiological saline solution (PSS). The abdominal aorta was also dissected, blotted dry, frozen quickly in liquid nitrogen and kept at −80°C until assayed. The heart, lungs and spleen were excised, blotted dry and weighed.

### Functional Studies of Resistance Arteries

Second-order branches of the mesenteric arteries (∼150–250 µm in diameter) from 10 control and 9 homo-βthal mice were carefully dissected from all adherent connective tissue of the intestine and bathed in a 5 mL organ chamber containing PSS of the following composition (in mmol/L) –118 NaCl, 25 NaHCO_3_, 4.7 KCl, 1.18 KH_2_PO_4_, 1.18 MgSO_4_, 2.5 CaCl_2_, 0.026 EDTA and 5.5 glucose – maintained at pH 7.4, warmed to 37°C and bubbled continuously with 12% O_2_, 5% CO_2_, and 83% N_2_. Each arterial segment was mounted and secured on 2 glass microcannulae with nylon ties, in a video-monitored pressure arteriograph system (Living Systems, Burlington, VT). The arteriograph system was placed on the stage of an inverted microscope (Nikon Eclipse TS100, Melville, NY) equipped with a video camera to monitor and measure vessel lumen diameter. The distance between moving cannulae was adjusted carefully to obtain horizontal vessel alignment without further stretching. Experimental assessment was started 1 hr after equilibration at 45 mmHg intraluminal pressure. Between each protocol described next, the system was washed out with PSS, re-equilibrated for 30 min, and resting lumen diameter was recorded. Vascular contractile reactivity was evaluated with dose-response curves to norepinephrine (NE, 10^−9^ to 10^−4^ mol/L, Sigma-Aldrich, St. Louis, MO). Endothelium-dependent and independent relaxation in response to cumulative concentrations of acetylcholine (ACh, 10^−9^ to 10^−4^ mol/L) and sodium nitroprusside (SNP, 10^−9^ to 10^−3^ mol/L), respectively, was determined by measuring the dilatory responses of vessels pre-contracted with a sub-maximal NE concentration (which produced 75 to 80% of maximal contraction, i.e. EC_75_–EC_80_). To study the contribution of NO to ACh-induced relaxation, concentration-response measurements were repeated after 30- min pre-incubation with *Nω*-nitro-L-arginine methyl ester (L-NAME, 10^−4^ mol/L). Relaxation was considered as a percentage of lumen diameter increased from resting diameter.

### Mechanical Studies of Carotid Arteries

The left carotid arteries of 13 controls and 11 homo-βthal mice were mounted in the pressure arteriograph system, adjusted to their *in vivo* length by displacing cannulae, and de-activated by perfusion with warmed and continuously-bubbled Ca^2+^-free PSS containing 10 mmol/L EGTA for 45 min at 45 mmHg. Intraluminal pressure was then raised 3 times from 3 to 140 mmHg to unbuckle the arteries. Vascular mechanics were evaluated by increasing intraluminal pressure from 3 to 180 mmHg by steps of 10 mmHg (except for the first step which was from 3 to 10 mmHg). For each pressure step, lumen diameter and wall thickness were measured by microscopy at 3 different positions along the vessel.

Carotid vascular wall cross-sectional area (CSA) was calculated as: (π/4)×(D_e_^2^–D_i_^2^), where D_e_ and D_i_ represent external and intraluminal diameters, respectively. The wall-to-lumen ratio was calculated as 2W/D_i_, where W is wall thickness. Circumferential strain (*ε*) was determined as (D_i_−D_i(0)_)/D_i(0)_, where D_i(0)_ is lumen diameter at 3 mmHg. Circumferential stress (σ) was given by (P×D_i_)/(2W) [18], where P is intraluminal pressure in dynes/cm^2^. Exponential fitting regression was performed on the stress-strain relationship of each vessel with the equation σ = σ_0_ e*^βε^*, where amplitude σ_0_ stands for stress at diameter D_i(0)_ and exponent *β* is the rate of increase of the stress-strain curve. This exponential model deduced the tangential elastic modulus (TEM) given by dσ/d*ε* = *β*σ. Therefore, *β* also represents the slope of the TEM-to-stress relationship and thus reflects the non-linear elastic properties of vascular wall components. Increases in *β* indicate amplified stiffness.

### Protein Expression Analysis

Frozen abdominal aortae (*n* = 6 per group) were pulverized separately in the presence of liquid nitrogen, and the powder was homogenized in lysis buffer (pH 8) containing 20 mmoles/L Tris-HCl, 150 mmoles/L NaCl, 2 mmoles/L EDTA and 0.5% Triton ×100 supplemented with protease inhibitor cocktail (No. P8340, Sigma-Aldrich) and phenylmethylsulfonyl fluoride (PMSF, 1 mmole/L final concentration). The samples were incubated for 30 min on ice and centrifuged at 4°C for 5 min. The supernatants were collected and stored at −20°C. Protein concentrations of the lysates were quantified by spectrophotometric Bradford protein assay (No. 500–0006, Bio-Rad, Mississauga, ON, Canada) with bovine serum albumin as standard. Protein lysates of bovine aortic endothelial cells (BAEC; 2 µg) served as positive controls. Equal amounts of aortic protein (20 µg/lane, except for BAEC control lane) were loaded, separated by sodium dodecyl sulfate polyacrylamide gel electrophoresis (SDS-PAGE) with the Novex 8% Tris-Glycine Pre-cast gel system (No. EC6018, Invitrogen, Burlington, ON, Canada) and transferred to a nitrocellulose membrane. Nonspecific sites were blocked with 5% non-fat powdered milk in phosphate-buffered saline (pH 7.4) with 0.1% Tween 20 (PBST) for 1 hr at room temperature. The membrane was incubated with primary mouse monoclonal antibody against endothelial NOS (eNOS) (1∶500; No. 610296, BD Transduction Laboratories, Mississauga, ON, Canada) for 2 hr at 4°C, washed 3 times (5 min each) in PBST and incubated with secondary antibody (peroxidase conjugated anti-mouse immunoglobulin 1∶5,000; No. A4416, Sigma-Aldrich) for 1 hr at 4°C. The membrane was then washed 3 times for 5 min in PBST, and the signal was detected by an ECL Western blotting system (No. RPN2132, Amersham, Baie d’Urfé, QC, Canada). Band intensity was quantified and expressed as a percentage of control band intensity. Constitutively-expressed protein GAPDH was the internal control.

### Histopathology Analysis

At the end of the mechanical experiments, the common carotid arteries from control and homo-βthal mice (*n* = 8 in each group) were fixed overnight in 10% phosphate-buffered formalin, embedded in paraffin and cut serially in 5-µm cross-sections. Tissue sections were stained with Verhoeff van Gieson for elastin. Images of all sections were taken under transmitted light at the same light intensity and exposure time settings. Six sections per vessel were analyzed. The relative elastin surface in the media was assessed semi-quantitatively by computer-assisted post-processing (Matlab software, version 7, release 14, MathWorks Inc., Natick, MA).

### Statistical Analyses

The data are presented as mean ± SEM. Statistics on the dose-response curves and mechanical parameters were analyzed by 2-way analyses of variance (ANOVA) for repeated measures, followed by the Student-Newman-Keuls test for multiple comparisons with post-hoc investigation. Histological elastin content and protein expression data were analyzed by unpaired Student’s *t* tests. *p*<0.05 was considered significant.

## Results

Fourteen-month-old homo-βthal mice were severely anemic and displayed significant splenomegaly, as described for human β-thalassemia major. Homo-βthal mice also had lower body weight and increased lung and heart weights normalized to body weight compared to the controls (Table 1). The 65% increment in normalized heart weight is indicative of cardiac hypertrophy, a phenotype consistent with severe clinical β-thalassemia [19].

**Table 1:** Characteristics of 14-month-old mice.

	Control	Homo-βthal
	(*n* = 13)	(*n* = 11)
BW (g)	32.7±1.3	28.1±0.8*
Hct (%)	41.9±1.1	27.0±1.2†
Heart weight to BW (mg/g)	4.5±0.2	7.3±0.3†
Lung weight to BW (mg/g)	4.6±0.2	5.9±0.1†
Spleen weight to BW (mg/g)	2.2±0.3	11.4±1.2†

Values are means ± SEM.

* *p*<0.05;

† *p*<0.001 vs. control mice. BW, body weight; Hct, hematocrit.

### Endothelial Vasomotor Function Impairment in Homo-βthal Mesenteric Arteries

**Figure 1:**
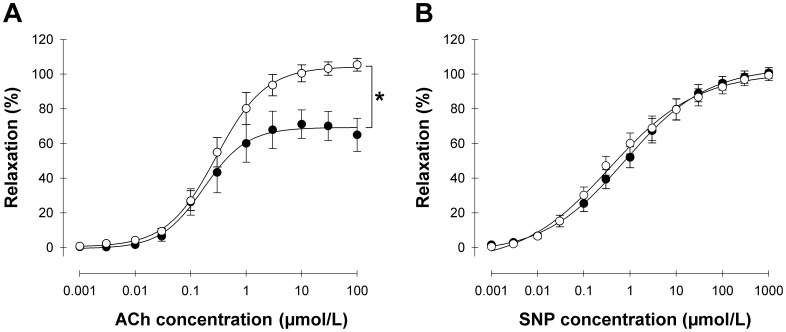
Vasodilatory responses of mesenteric resistance arterioles to acetylcholine (ACh) (A) and sodium nitroprusside (SNP) (B), in control (○; *n* = 10) and homo-βthal (•; *n* = 9) mice. Relaxation responses are expressed as a percentage increase in lumen diameter after norepinephrine pre-contraction. Data are means ± SEM. ^*^ *p*<0.05.

**Figure 2:**
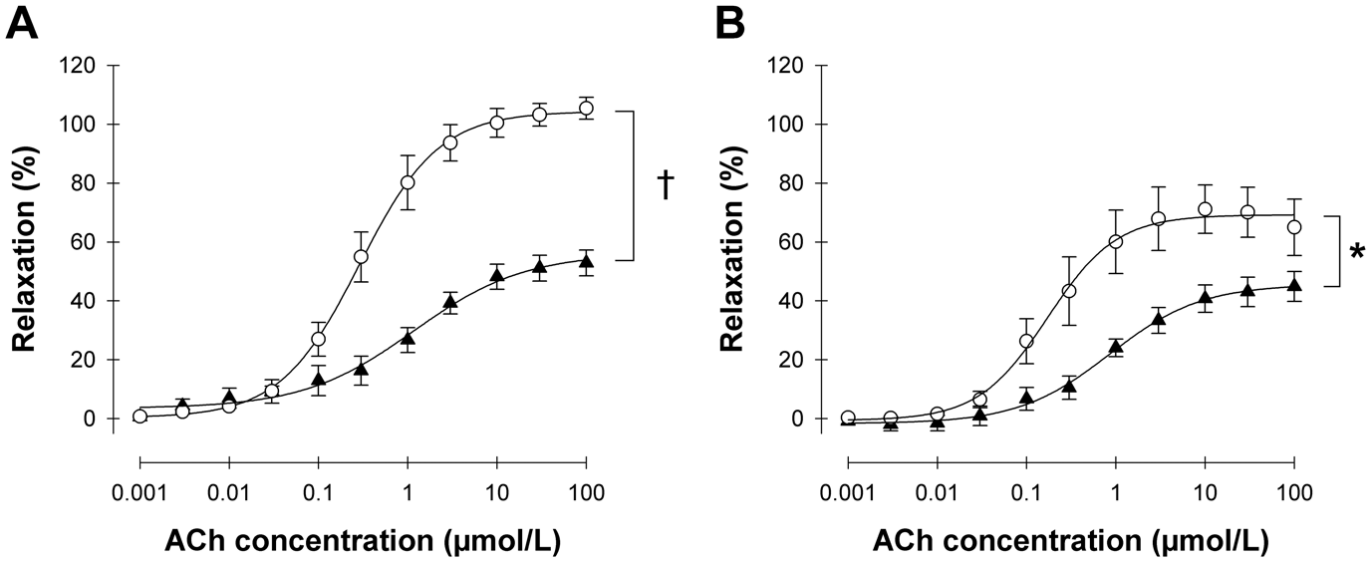
Endothelium-dependent vasodilatory responses of mesenteric resistance arterioles from control (*n* = 10) (A) and homo-βthal mice (*n* = 9) (B) to acetylcholine (ACh) in the absence (○) or presence ( ▴**) of L-NAME.** Relaxation responses are expressed as a percentage increase in lumen diameter after norepinephrine pre-contraction. Data are means ± SEM. ^*^ *p*<0.05 and ^†^ *p*<0.001.

Vascular function was assessed in isolated mesenteric arteriole preparations from 14 month-old control (*n* = 10) and homo-βthal (*n* = 9) mice. NE-mediated vascular contraction was similar in both groups (data not shown). Endothelium-dependent vasodilation in response to ACh, however, was significantly impaired in the arterioles of homo-βthal mice with a maximum response of 71.1±8.2% compared to 105.4±3.7% for control mice (Figure 1A). In contrast, endothelium-independent relaxation stimulated by SNP was almost identical in homo-βthal and control arterioles (Figure 1B). Although inhibition of NO synthesis by L-NAME reduced ACh-induced vasodilation in arterioles of both mouse groups, this suppressive effect was significantly lower in homo-βthal mice (*p*<0.05) than in the controls (32.3±8.7% vs. 55.5±6.1% inhibition of the maximum response, respectively, Figure 2). Therefore, our results indicate preserved smooth muscle cell function in β-thalassemic mice with endothelial vasodilatory dysfunction and decreased NO bioavailability.

### Overexpression of Vascular eNOS Protein in Homo-βthal Mice

**Figure 3:**
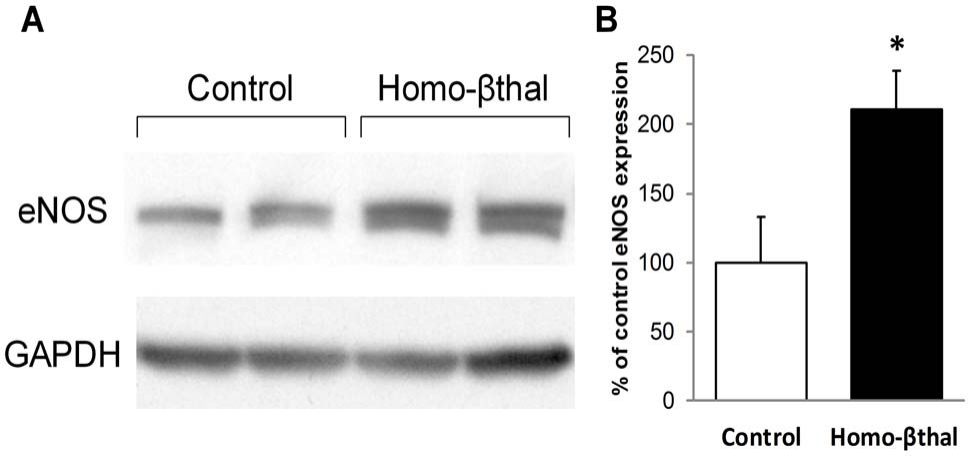
eNOS protein expression in aortae of control and homo-βthal mice. (A) Representative Western blotting, and (B) densitometric analysis of eNOS protein expression (*n* = 6 mice per group). Measurements are expressed as % of eNOS bands in aortae of control mice. Data are means ± SEM. ^*^ *p*<0.05.

By aortic Western blot analysis, we assessed whether the alterations in endothelium-dependent vasodilation were secondary to changes in eNOS expression. Homo-βthal mice presented a 2-fold increase in eNOS expression versus the controls (*p*<0.05, Figure 3). Thus, the afore-mentioned endothelial dysfunction and decrease in NO bioavailability occurred despite eNOS up-regulation.

### Alterations in Carotid Artery Vascular Mechanics in Homo-βthal Mice

**Figure 4:**
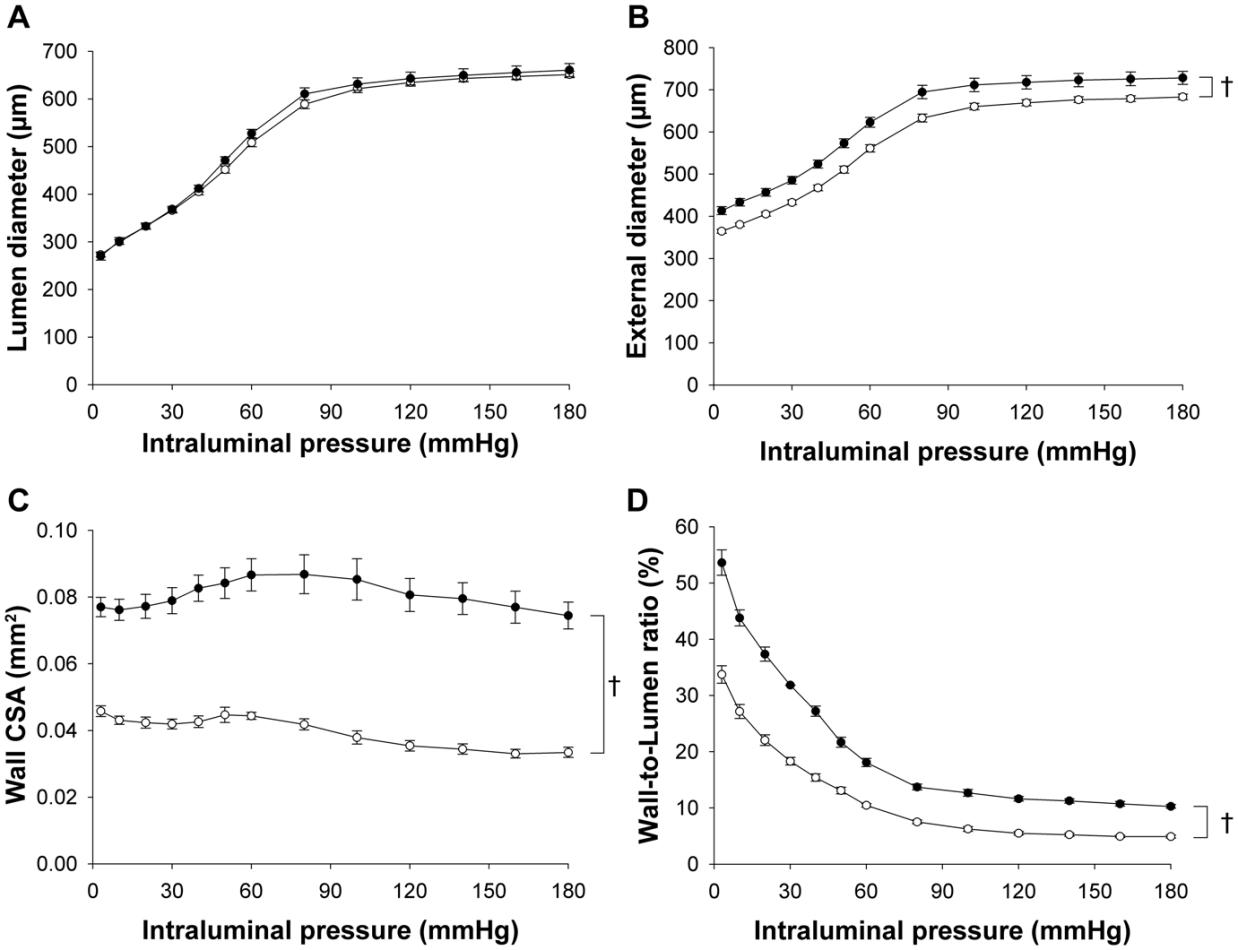
Comparison of structural characteristics in common carotid arteries from control (○; *n* = 13) and homo-βthal mice (•; *n* = 11). (A) Luminal diameter, (B) external diameter, (C) wall cross-sectional area (CSA), and (D) wall-to-lumen ratio versus intraluminal pressure. Data are means ± SEM. ^†^ *p*<0.001.

**Figure 5:**
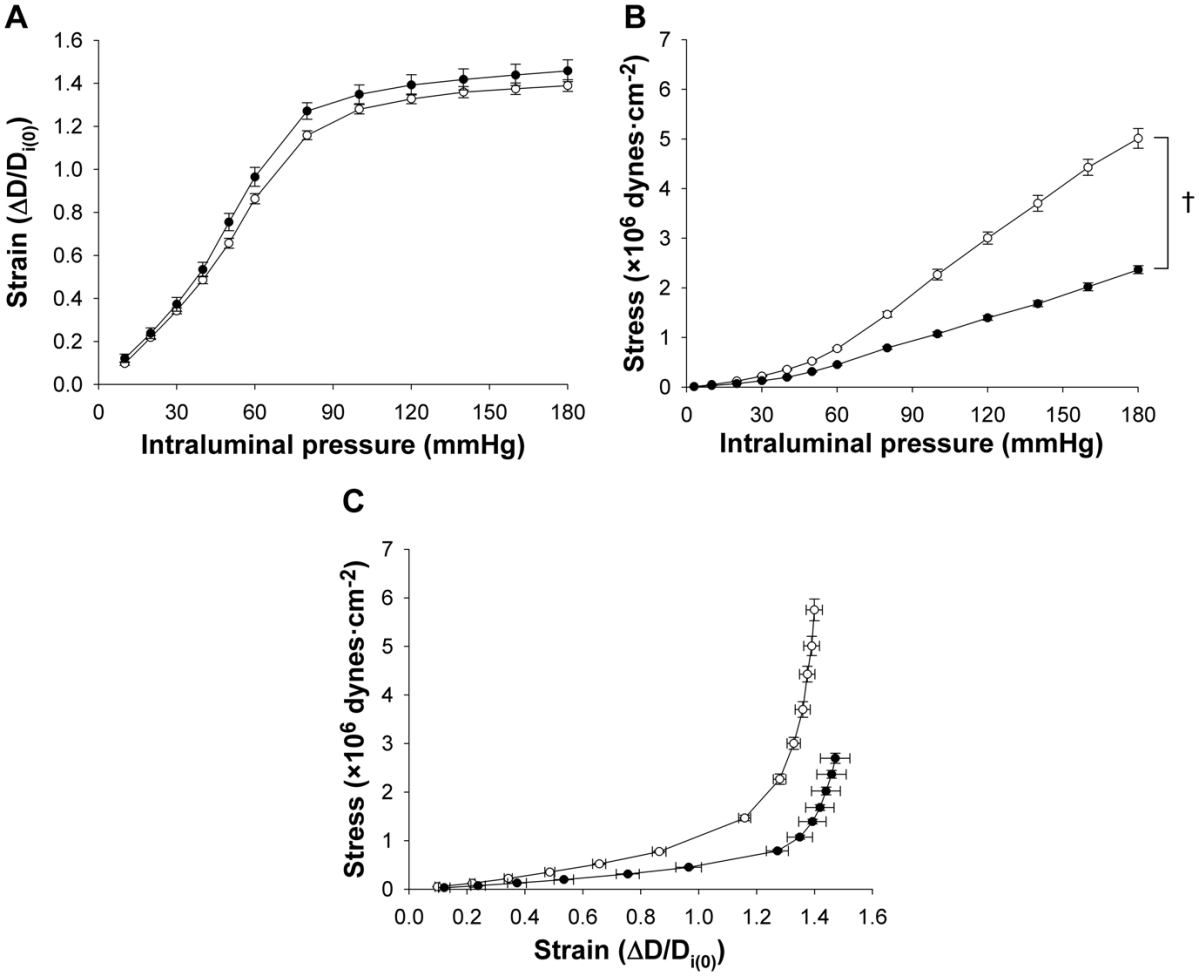
Comparison of mechanical parameters in common carotid arteries from control (○; *n* = 13) and homo-βthal mice (•; *n* = 11). Circumferential strain-intraluminal pressure (A), circumferential stress-intraluminal pressure (B), and stress-strain (C) relationships. Data are means ± SEM. ^†^ *p*<0.001. The curve in panel C exhibits a rightward shift in the homo-βthal group, as shown by a significant decrease in *β* (*p*<0.01) and the slope of the tangential elastic strain modulus-to-stress relationship.

**Figure 6:**
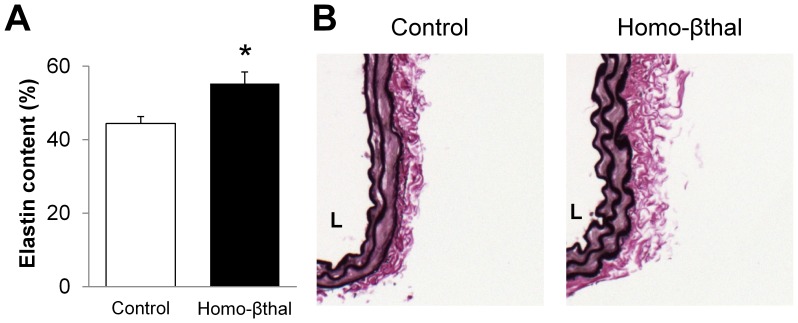
Histological analysis of elastin content in media (A) and representative microscopy images of Verhoeff van Gieson-stained sections (B) of the left common carotid arteries in control (open bars) and homo-βthal mice (closed bars, *n* = 8 per group). Data are means ± SEM. ^*^ *p*<0.05. L, vascular lumen.

The mechanical properties of carotid arteries in control (*n* = 13) and homo-βthal (*n* = 11) mice were studied by correlating passive changes in vascular diameters with step-wise increments in intraluminal pressure. The carotid arteries of both groups displayed similar increases in luminal diameter in response to augmented intraluminal pressure from 3 to 180 mmHg (Figure 4A). Carotid external diameters in homo-βthal mice were significantly larger at all intraluminal pressures (Figure 4B), reflecting vascular wall thickening and significantly higher wall CSA (Figure 4C). The increased wall-to-lumen ratios in homo-βthal mice compared to the controls are indicative of carotid artery hypertrophic remodeling (Figure 4D). While circumferential wall stresses were significantly lower in homo-βthal carotid arteries (Figure 5B) compared to the controls, circumferential strain values were similar overall in both groups (Figure 5A). Consequently, the non-linear circumferential stress-strain curve shifted rightward in the homo-βthal group (Figure 5C), which proves softening of the arteries in comparison to the controls. This was confirmed by lower mean values of *β* (slope of the TEM-to-stress relationship) in homo-βthal mice (3.11±0.08, *p<*0.01) compared to the controls (3.43±0.05). Softening of carotid artery structural components was further proven by semi-quantitative histological analysis with Verhoeff van Gieson staining, revealing that the relative surfaces of elastin in the media of homo-βthal carotids were significantly increased compared to the controls (Figure 6A and B, *p*<0.05).

## Discussion

For the first time, the present study investigated the endothelium-mediated NO vasodilation response in resistance arteries as well as the mechanical properties and structure of the common carotid arteries in β-thalassemic mice. Our results confirmed that: 1) β-thalassemic mice, homozygous for deletion of the murine β-major gene, display significant impairment of resistance arteriole endothelial-dependent vasodilator function in conjunction with heightened levels of eNOS expression and preserved smooth muscle cell reactivity to NO; and 2) the carotid arteries of β-thalassemic mice are characterized by wall thickening and softening of structural components with increased elastin content.

### Endothelium-dependent Vascular Reactivity

Previous clinical studies have suggested that endothelial dysfunction in the conduit arteries of β-thalassemic patients is due to decreased NO bioavailability [10]. Our results on resistance arterioles are consistent with this possibility, since we observed attenuated endothelium-dependent vasodilation in response to ACh in β-thalassemic mice. NO bioavailability is determined by a balance between NO production and consumption. Our results present, for the first time, that endothelial dysfunction in β-thalassemia occurs despite increased eNOS expression levels. This suggests either impaired NOS activity or increased NO scavenging. β-thalassemia is associated with chronic intravascular hemolysis that is known to induce de-compartmentalization of two main erythrocyte components into plasma: hemoglobin and arginase [20]. Cell-free plasma hemoglobin reacts rapidly with, and deactivates, NO [21]. It has been shown to elicit a significant decrease of NO bioavailability *in vivo* and endothelial dysfunction [22] by limiting NO diffusion from the endothelium to smooth muscle cells and, consequently, inhibiting vasodilation. While this mechanism of NO-scavenging is likely to contribute to further declines of NO bioavailability in β-thalassemia *in vivo*, it does not apply to the current experimental *ex vivo* setting of isolated mesenteric arterioles. However, spontaneous release of erythrocyte arginase during intravascular hemolysis may limit cellular availability of the eNOS substrate for NO synthesis, L-arginine, resulting in deficient NO production. In fact, studies of patients affected by sickle cell disease, another hemoglobinopathy inducing chronic hemolytic anemia, have shown increased intravascular hemolysis-related plasma arginase activity [20]. In β-thalassemia patients, heightened erythrocyte arginase activity strongly correlating with plasma arginase activity has also been reported [23]. In β-thalassemic arteries, decreased plasmatic L-arginine should lead to reduced cellular uptake of the substrate and consequent alteration of NO production despite increased eNOS expression.

Moreover, further diminution of NO bioavailability may result from increased oxidative stress. A common mechanism of endothelial dysfunction in hemolytic anemias has been suggested by studies of β-thalassemia [24], sickle cell disease [25] and mouse models of severe hemolysis [26] reporting the increased oxidizing potential of plasma, possibly generated directly by cell-free hemoglobin, heme and heme-iron [27], which might damage and activate the endothelium by evoking oxidative injury. In addition, enzymatic pathways of reactive oxygen species (ROS) overproduction – of superoxide, for example – by activating vessel wall xanthine oxidase and NADPH oxidase, have been postulated to play a role in the generation of oxidative stress in both β-thalassemia and sickle cell disease [20]. Finally, the paradoxical decrease in NO production, even though eNOS levels were elevated, might reflect an eNOS uncoupling mechanism. The possible decrease in L-arginine availability might culminate in eNOS dimer disruption or uncoupling, causing superoxide instead of NO production [28], [29] and further adding to the reduction of NO bioavailability as well as increased oxidative stress in β-thalassemia.

### Endothelium-independent Vascular Reactivity

In addition, a discrepancy with respect to previous clinical investigations was reported concerning endothelium-independent vasodilation in β-thalassemia [10], [11], [30]. Indeed, our findings confirm preserved endothelium-independent vasodilation in response to SNP, a direct donor of NO metabolized by smooth muscle cells. This important insight indicates that resistance arteriole vasodilatory impairment occurs in the absence of vascular smooth muscle cell dysfunction in NO signaling at the level of the guanylate cyclase-cGMP system. Therefore, vascular dysfunction in our β-thalassemic mice does not seem to be associated with NO resistance or lack of responsiveness.

### Carotid Artery Structure and Mechanical Properties

The β-thalassemia major mouse model not only presented resistance arteriole endothelial-mediated vasomotor dysfunction but also displayed altered carotid artery structure with changes in the mechanical properties of wall components. Our study demonstrated the development of carotid artery wall hypertrophic remodeling characterized by an increase in both total wall thickness and wall-to-lumen ratios. This wall thickening was coupled with growth of medial elastin content within the β-thalassemic carotid artery wall.

Clinical studies of β-thalassemia major patients have reported increased arterial stiffness of the carotid arteries [11], [31], brachial arteries [11], [12], ascending [14] and abdominal aortae [32]. The explicative mechanisms of discrepancy between these clinical and our murine β-thalassemia vascular mechanical results remain unknown and require further investigation. Nevertheless, clinical studies have demonstrated that stiffness of the abdominal aorta in regularly-transfused β-thalassemia major patients was increased in relation to liver iron concentration, a reliable indicator of tissue iron-loading [32]. Iron overloading has been known to augment systemic and vascular wall ROS generation, which might contribute to the development of vascular injury [33]. These findings led us to hypothesize that the β-thalassemic vasculature remodels differently in the absence of palliative transfusion treatments, evoking iron-overloading.

Anemia-induced hypoxia and oxidative damage might have contributed to arterial structural changes in β-thalassemic mice. In fact, hypoxia is known to promote vascular cellular proliferation and matrix synthesis by inducing a variety of genes in endothelial cells, eliciting the production of mitotic factors, such as platelet-derived growth factor B, insulin-like growth factor and vascular endothelial growth factor [34]. In addition, studies have indicated that ROS might provoke vascular structural and functional alterations by activating several intracellular signaling cascades, namely, extracellular signal-regulated kinases, mitogen-activated protein kinases, tyrosine kinases, protein tyrosine phosphatases and transcriptional factors, such as nuclear factor kappa B and activated protein 1 [35].

In conclusion, our study of non-transfused β-thalassemic mice provided evidence of functional changes in endothelium-dependent vascular responses characterized by dysregulation of endothelial NO production and consequent NO bioavailability. In addition, endothelial dysfunction of resistance arterioles occurred concomitant to structural alterations of the carotid artery wall and modification of the mechanical properties of wall components. Hemolysis likely contributed to the pathophysiology of both endothelial dysfunction as well as vascular structural and mechanical changes in β-thalassemia. Treatments targeting hemolysis-related complications, namely, erythrocyte de-compartmentalization of hemoglobin and arginase, may represent potential therapeutic strategies. Future investigations with this mouse model should provide a better understanding of underlying molecular processes in the pathogenesis of β-thalassemia major vasculopathy.
